# Internationalization and Collaboration in Colombian Psychology During 2014–2023: A Bibliometric Analysis

**DOI:** 10.11621/pir.2024.0404

**Published:** 2024-12-01

**Authors:** Claudio Bustos Navarrete, Lina Villota Castillo, Astrid Sarmiento Quiñones, Ignacio Rojas Rodríguez, Lina Sandoval Moreno

**Affiliations:** a University of Concepción, Chile; b Virginio Gómez Professional Institute, Concepción, Chile; c University San Sebastián, Concepción, Chile; d University of Valle, Cali, Colombia

**Keywords:** bibliometrics, psychology, Colombia, scientific cooperation, internationalization

## Abstract

**Background:**

Bibliometric studies are essential for understanding the development of a discipline and thus establishing policies to promote evidence-based science. In the case of Colombian psychology, no studies have yet considered the productivity, collaboration, and internationalization of this discipline jointly. In this context, the role of Ministerio de Ciencia, Tecnología e Innovación (Minciencias) research groups in promoting collaboration within Colombia is particularly significant.

**Objective:**

To analyze the scientific productivity, degree of internationalization, and collaboration in Colombian psychology from 2014 to 2023.

**Design:**

A bibliometric study was conducted using data from Web of Science, Scopus, and SciELO, employing bibliometric indicators. A total of 4,196 Colombian psychology articles from 2014 to 2023 were analyzed, employing various indicators to assess productivity, internationalization, and collaboration.

**Results:**

The period was characterized by a sustained increase in productivity. An increase in internationalization was evident, as shown by a greater number of articles written in English and published in foreign journals, along with growing international collaboration, primarily with Spain and the United States. While Colombian-led research has increased, publications with foreign leading authors receive a substantially higher number of citations.

**Conclusion:**

This is the first study to incorporate Minciencias groups into the evaluation of research productivity within Colombian psychology. The study suggests that researchers are adapting to Minciencias’s evaluation criteria, with a focus on collaboration and high-impact publications. Strengths include using multiple databases and rigorous data cleaning. Future research can explore international comparisons and the impact of internationalization on research focus.

## Introduction

Scientific collaboration is fundamental for the development of any discipline, as it allows complementing strengths and weaknesses of individuals and groups, and generating a critical mass of researchers specialized in specific topics (Aleixandre et al., 2013). Bibliometrics allows the measurement of researchers’ collaboration and productivity. This discipline organizes scientific information from various documentary sources for subsequent quantitative and qualitative analysis. Bibliometric analysis enables the identification of fields of production, collaboration networks, and author productivity, among other aspects (Gallegos et al., 2020).

Diffierent disciplines exhibit variations in the forms and extent of their scientific collaboration, which are reflected in their bibliometric indicators (Wagner et al., 2017). The relationship between collaboration and productivity is complex, varying across disciplines, between basic and applied levels, and within the context of domestic versus international collaboration. International collaboration generally results in a higher number of citations, with large multinational projects generating even more citations; however, this relationship depends on the discipline, being stronger in the life sciences (Glänzel & de Lange, 2002). Psychology has shown a low level of international collaboration compared to other sciences. In 1997, approximately 35% of articles involved international cooperation, increasing to 45% in 2012. By comparison, the discipline with the highest level of cooperation is astronomy, which had a cooperation rate of 68% in 1997, rising to 83% in 2012 (Coccia & Wang, 2015).

The global development of productivity in psychology is reflected in the growth of psychology in Spanish-speaking countries. At the beginning of the 21st century, around 500 scientific psychology journals were published in Latin America, covering various branches of psychology (Buela-Casal & López, 2005). Gallegos et al. (2020) conducted a meta-bibliometric review covering 1977–2015, based on 81 bibliometric studies from Ibero-America, which includes Latin America, Spain and Portugal, published in 15 regional journals. They concluded that traditional areas, such as clinical psychology, organizational psychology, and social psychology, predominated over “emerging” fields such as forensic psychology, consumer psychology, and traffic psychology and road safety. Spanish (88%) predominates over Portuguese (6%) and English (6%), with Spain (55%) leading the Ibero-American bibliometric research landscape.

### Historical Development of Psychology in Colombia

Psychology in Colombia has made significant strides since its inception in 1930, advancing in both academic and applied fields. Like Latin American psychology, Colombian psychology has a scientific orientation taught in academic institutions, which includes the development of theses and participation in master’s and doctoral programs (Ardila, 2004). In the applied domain, Ardila & Castro (1973) highlight its important role in Colombian society, emphasizing its contributions to the effective use of human resources through personnel selection processes, the advancement of the educational system, and the comprehensive study of motivation and poverty. Today, the discipline continues to generate academic and scientific knowledge that addresses the challenges faced by Colombian individuals and communities (Vesga, 2021).

The history of Colombian psychology can tentatively be divided into three main stages: (a) foundation and expansion; (b) institutionalization; and (c) consolidation. The first stage, foundation and expansion, covers the period from 1930 to 197. It formally began at the university level with the establishment of the Psychotechnical Section at the National University in 1939. This evolved into the Institute of Applied Psychology in 1948, later becoming the Faculty of Psychology in 1957, and finally the Department of Psychology in 1965, solidifying its position as the principal training center for psychologists in the country. In the academic field, significant challenges were encountered, such as the lack of translations of key theoretical works, which necessitated the long-term incorporation of foreign professors (Ardila, 1970, 1999, 2004).

During the 1950s and 1960s, the discipline began to expand into applied areas, marked by the establishment of the Colombian Federation of Psychology in 1954. This organization aimed to regulate the profession and broaden its scope beyond the academic sphere, extending into industry and psychological care. Another significant development during this period was the launch of the Revista de Psicología [Psychology Magazine] in 1956, which published chronicles and specialized bibliographies, including multilingual reviews, thereby contributing to the international dissemination of the field. Despite these advancements, this initial stage was characterized by limited international engagement, with most publications being of national origin (Ardila, 1970, 1999, 2004).

From 1970 onward, experimental psychology gained prominence, coinciding with the establishment of new university programs at institutions such as Universidad de Los Andes, Universidad del Norte, and Universidad Católica de Colombia (Ardila, 1999). In 1974, under the leadership of Rubén Ardila, the so-called Bogotá Model was introduced—a generalist training system inspired by the American Boulder model. This model combined professional and academic training, preparing graduates to work across various fields of applied psychology. It represented a significant milestone, solidifying Colombian psychology as a discipline that contributed meaningfully to both theoretical development and practical application in diverse sectors (Cudina & Ossa, 2019).

The second stage, characterized by institutionalization, spans the 1980s and 1990s. It began with the enactment of Law 58 in 1983, which regulated the psychology profession, and the establishment of the psychology program at Pontificia Universidad Javeriana of Cali in 1984 (Ardila, 1999). During this period, the Colombian Society of Psychology, founded in 1979 to replace the Colombian Federation of Psychology, played a crucial role. It united the majority of Colombian psychologists until 2014 and promoted theoretical and methodological advancements through congresses (Cudina & Ossa, 2019). The 1990s ushered in further changes with the 1991 Constitution and Law 30 of 1993, which, by establishing university autonomy, facilitated the creation of new academic programs. This institutionalization process culminated in 2004 with the launch of the first doctoral program at Universidad del Valle.

The third phase of consolidation began in the 2000s. In 2004, the first doctoral program was established at Universidad del Valle, alongside the emergence of two significant organizations: the Colombian Association of Psychology Faculties (Ascofapsi), which focuses on ensuring the quality of psychology education, and the Colombian College of Psychologists (Colpsic), dedicated to overseeing professional practice (Cudina & Ossa, 2019; López-López et al., 2022). In terms of productivity, the 2000s were marked by limited collaboration between national and foreign authors. Although there was increased editorial acceptance of both national and international articles, this did not necessarily translate into collaborative research efforts (Jaraba-Barrios et al., 2011).

### The Current State of Psychology in Colombia: Training, Research, and Productivity

According to López-López et al. (2022), the generation of knowledge in psychology, as well as its dissemination, depends on a *knowledge ecosystem* involving factors such as the infrastructure of higher education institutions, public science and technology policies, and national and international collaboration networks. In the case of Colombia, although academic productivity has increased, driven by the availability of graduate programs, it has primarily focused on national journals. Dissemination in high-impact international journals remains limited, restricting the global visibility of the knowledge produced. This ecosystem also faces challenges due to declining investment in research and development, which decreased from .3% of GDP in 2014 to .23% in 2018, well below the levels of other Latin American countries. Additionally, pressures to meet indexing and accreditation indicators have fostered a publication culture that, while increasing the quantity of outputs, often compromises quality and international visibility. Nevertheless, efforts to strengthen scientific cooperation networks and consolidate Colombian psychology within the regional and international context are noteworthy.

In Colombia, psychology as a discipline has been consolidated in the 2020s. This is evidenced by four indicators: (a) the presence of many undergraduate and graduate programs; (b) the formation of research groups in the field of psychology; (c) the inclusion of Colombian psychology journals in indexed bibliographic databases, such as Scimago Journal Rank and Web of Science; and (d) the increase in bibliographic productivity (Puche-Navarro et al., 2022).

Psychology experienced an exponential increase in academic programs between 1970 and 2020. These programs were developed independently by universities and accredited higher education institutions, offered in face-to-face, distance, and virtual modalities. In 1973, only three programs existed nationwide, but by 2022, there were more than 100 programs and 16 doctoral programs (Puche-Navarro et al., 2022). A descriptive study conducted at the end of 2022 identified 132 active undergraduate programs, with the majority concentrated in the private sector (81%) and delivered in face-to-face modality (92.4%). The current geographic distribution of programs highlights a shifttowards decentralization, with higher education institutions now present across the country, whereas previously, training was concentrated in the capital (Torres, 2023).

Noteworthy is the growth in doctoral training in Colombia, which began with the first doctoral program at Universidad del Valle in 2004 and expanded to 16 programs by 2020. The contribution of postgraduate research to the overall productivity of Colombian psychology is noteworthy. A study examining the productivity of 13 doctoral departments found a positive correlation between the number of graduate theses advised in each department and publication in national journals (rho = .61). Additionally, a moderate correlation was observed between the number of theses and publication in international journals (rho = .41). However, discrepancies were noted between productivity reported in the CvLAC system (Curriculum Vitae de Latinoamérica y el Caribe [Curriculum Vitae from Latin America and the Caribbean]) and Scopus. These diffierences could be attributed to a limited publication culture within graduate programs and a focus on local dissemination, often at the expense of publishing in internationally indexed journals (López-López et al., 2022).

The consolidation of research groups in psychology is evident in the significant number of groups classified in superior quality categories and their ability to maintain their classification or advance within these categories. In Colombia, Law 1951 of 2019 established the Ministry of Science, Technology, and Innovation (Minciencias) to promote national scientific development. The ministry oversees research and development, identifying the institutions and individuals involved, the products they develop, and their modes of interaction. It primarily funds recognized institutions and groups rather than individual researchers (Ministerio de Ciencia, Tecnología e Innovación [Ministry of Science, Technology and Innovation], 2020a).

Research groups recognized in Colombia are evaluated according to the Model for the Measurement of Research, Technological Development or Innovation Groups and Recognition of Researchers of the National System of Science, Technology, and Innovation. This evaluation includes a collaboration profile with two key indicators: *cohesion*, measuring internal group collaboration; and *cooperation*, assessing intergroup collaboration through co-authorship. These two indicators are part of the Group Indicator, a polynomial that considers eight indicators, in which the cooperation indicator has a weight 4 times higher than the cohesion indicator. Publications in Web of Science and Scopus are prioritized for their visibility and impact (Ministerio de Ciencia, Tecnología e Innovación [Ministry of Science, Technology and Innovation], 2020b).

As of May 15, 2024, there were 160 registered Minciencias psychology groups throughout Colombia. According to the Minciencias evaluation model, these groups were classified into five categories, based on various criteria, including their productivity and level of collaboration, which are A1, A, B, C, and Recognized, ranked from highest to lowest. Of the 160 groups, 36 groups are category A1, 39 groups are category A, 36 groups are category B, 39 are category C, and 10 are in the Recognized category (Ministerio de Ciencia, Tecnología e Innovación Colombia [Ministry of Science, Technology and Innovation Colombia], 2023). Notably, in 2011, only one psychology group held the A1 category, but by 2024, 36 groups had achieved this status. Furthermore, 31% of the groups were promoted, 56% retained their category, and only 9% were demoted (Puche-Navarro et al., 2022).

Another indicator of consolidation is the presence of Colombian psychology journals in international indexed databases. As of October 2024, a total of 14 Colombian journals are registered in Scopus under the psychology category: (a) *International Journal of Psychological Research;* (b) *Universitas Psychologica [Psychological Universities];* (c) *Revista Latinoamericana de Psicología [Latin American Journal of Psychology];* (d) *Acta Colombiana de Psicología [Colombian Psychology Act];* (e) *Avances en Psicología Latinoamericana [Advances in Latin American Psychology];* (f) *Revista CES Psicología [CES Psychology Magazine];* (g) *Suma Psicológica [Psychological Sum];* (h) *Revista Colombiana de Psicología [Colombian Journal of Psychology];* (i) *Revista Latinoamericana de Ciencias Sociales, Niñez y Juventud [Latin American Journal of Social Sciences, Children and Youth];* (j) *Revista Criminalidad [Crime Magazine];* (k) *Nómadas [Nomads];* (l) *Psicogente [Psychopeople];* (m) *Revista Guillermo de Ockham [William of Ockham Magazine];* and (n) *MedUNAB.* The first three journals are included in the Social Sciences Citation Index of Web of Science, while the first four appear in the Emerging Sources Citation Index. The latter index also includes the journal *Pensando Psicología [Thinking Psychology],* which is not listed in Scopus.

Finally, there has been a significant increase in overall productivity in psychology. Following the modification of its National Science and Technology System (Law 1289 of 2009), Colombia has promoted the evaluation of research in public and private institutions through productivity and quality indicators, aligning with global rankings such as the Shanghai Ranking, the Ibero-American SIR, and the Colombian Atlas of Science. This shiftmarks the formal integration of Colombian science into the international citation-based evaluation system, driving strategies to enhance its performance in these metrics (Romero-Torres et al., 2013).

### Collaboration and Internationalization in Bibliometric Studies of Colombian Psychology

Bibliometric studies have played a crucial role in analyzing the growth and development of psychology in Colombia, particularly from 2010 onwards. Numerous studies have examined the productivity of Colombian psychology journals (Aguilar & Aguado-López, 2018; Ávila-Toscano et al., 2014; Ávila-Toscano & Marenco-Escuderos, 2016; Morgado-Gallardo et al., 2018; Ravelo-Contreras et al., 2016, 2020; Rivera-Garzón, 2008; Salas et al., 2018, 2019). Also, specific subfields, such as educational psychology (Restrepo et al., 2015) and consumer psychology (Maldonado & Pérez-Acosta, 2020) have been studied through bibliometric analyses.

Two main topics in bibliometric research on Colombian productivity in psychology have been the language of publication and collaboration. Spanish has predominated in the publications. However, over the years, Colombian journals have incorporated the publication of articles in English and some in Portuguese (Ávila-Toscano & Marenco-Escuderos, 2016; Ravelo-Contreras et al., 2016). An increase in the level of collaboration in publications is observed over the years. In the case of the *Revista Acta Colombiana de Psicología [Acta Colombiana de Psicología Magazine],* an increase is observed from 2.64 authors per article in 2010–2014 (Ravelo-Contreras et al., 2016), to 3.32 in 2015–2019 (Ravelo-Contreras et al., 2020). In international collaboration, it was found that between 2015 and 2019, 47% of the articles in psychology in Colombian publications were produced by researchers from several countries (León-Cano et al., 2022). Similarly, in the journal *Suma Psicológica [Psychological Sum],* there was an increase from 2.3 authors per article in 1994–1997 to 3.9 in 2014–2017 (Morgado-Gallardo et al., 2018).

Internationalization is understood as the process of integrating global, intercultural, and international dimensions into the purpose, function, and delivery of tertiary education (Knight, 2003). Aguado-López et al. (2017) indicate that, in the case of publications, the percentage of articles published in foreign journals, the percentage of articles co-authored with foreign researchers and the impact abroad—the latter understood as citations received in foreign publications—are used as indicators of internationalization. The importance of internationalization, according to Nassi-Calò (2017), lies in reaching a larger and more demanding audience. This is achieved with the improvement of journals, which attracts better articles and readers and authors from other parts of the world, reinforcing the virtuous circle of larger audience / better articles. However, internationalization can bring negative consequences: by joining foreign research currents, local researchers may abandon lines of research associated with national phenomena and, therefore, stagnation may occur in the scientific development of the country (Chinchilla-Rodríguez et al., 2015).

Regarding the internationalization of publications in Colombian psychology, León-Cano et al. (2022) found that between 2015 and 2019, 47% of the articles in psychology with a Colombian affiliation published in Scopus were produced by researchers from several countries. Cudina & Ossa (2016) analyzed the 100 articles with the highest impact in Colombian psychology from 1972 to 2016, considering those with the highest number of citations registered in Scopus and Web of Science. They found that 81% of these papers were published in international journals and 77% were published in the English language. The same study indicated that the main international collaboration was with authors from the United States and, in second place, Spain, noting that the network between the Colombian academic community and Latin American countries was very incipient. The strength of collaboration with certain countries depends on the area of research; in the specific case of health psychology, the relationship with Spain stands out, sharing 16% of the articles in this area (Salamanca-Camargo & Tovar-Gamboa, 2022).

### Research Gap and Objectives

Despite the existence of multiple bibliometric studies of Colombian psychology, there has been no systematic study that considers the diffierent levels of organization of its productivity. This includes individual researchers, their affiliations, and the structure of the national and international collaborative network. Although Minciencias promotes research collaboration, there have been no systematic studies of its relationship to productivity in psychology; there is only one article specifically addressing the gender gap at a university in the Cauca Valley (Caicedo-Ortiz et al., 2021). Furthermore, efforts to evaluate the internationalization of Colombian psychology are incipient, have focused on a limited set of articles and databases, and have not studied the degree of collaboration between domestic and international communities. Finally, most studies consider only a specific group of journals and, at most, a bibliographic database, which generates an important bias when attempting to describe the productivity of Colombian psychology. Therefore, the general objective of the present research is to analyze the scientific productivity, degree of internationalization and collaboration of Colombian psychology between 2014 and 2023, through the study of the publications indexed in the bibliographic databases Web of Science, Scopus and SciELO. For this purpose, the specific objectives will be: (a) to describe the scientific productivity of Colombian psychology during 2014–2023, (b) to analyze the degree of collaboration between authors, affiliations, Minciencias groups, and countries in Colombian psychology during 2014–2023, (c) to analyze the degree of internationalization of Colombian psychology according to language, country of the journal, and international co-authorship during 2014–2023.

## Methods

This study uses a bibliometric design, which analyzes scientific production through mathematical and statistical methods. Productivity was described through bibliometric indicators, providing quantitative and qualitative information related to the scientific articles studied (Gallegos et al., 2020). Both the process of searching for articles and the analysis were generally based on the approach described by Uribe-Bahamonde (2022) in a study of Chilean productivity in psychology. This involves an initial search process, followed by a data filtering and normalization process, and concluding with data analysis.

As the main bibliometric indicators of productivity, we considered the number of articles per year, their language, the authors’ affiliations, and their affiliation countries, authors’ gender, distribution of productivity per author, productivity of the main national and foreign affiliations, as well as the productivity of Minciencias groups and Colombian affiliations. With respect to collaboration, various indicators were used to measure collaboration, considering the co-authorship of multiple researchers, affiliations, Minciencias groups, and countries. Finally, with respect to internationalization, productivity in foreign journals, number of citations, and language of publication were analyzed. The details of the calculation of the indicators are presented in *Table 1*.

**Table 1:** Bibliometric Indicators

Indicator	Description
Average annual growth rate	Let *k* be the number of periods and *x_i_* be the value of the indicator for the *i*-th period. The average annual growth is:
	$\text{AAGR}=\frac{1}{\text{k}-1}\sum_{i=1}^{k}\frac{x_{i}-x_{i-1}}{x_{i-1}}$
Lawani Collaboration Index	Indicates the average number of entities (authors, affiliations, countries) per article. Let *N* be the total number of articles, and *n_j_* the number of papers with *j* entities. The Lawani index is (Lawani, 1980):
	$\text{LCI}=\frac{\sum_{j=1}^{\text{A}}jn_{i}}{N}$
Subramanyam index total and by year	Proportion of articles with 2 or more entities (authors, affiliations, countries).
	$SI=\frac{\sum_{j=2}^{\text{A}}n_{j}}{N}$
Minciencias’s Cooperation indicator (Icoop)	Let *G* be the number of Minciencias groups related to *N* articles. The index *Icoop* is:
	$\mathrm{ICoop}=\frac{G}{N}-1$
Minciencias’s cohesion indicator (IC)	For a given Minciencias group, let *N* be the number of articles made by the group. A*_i_* is the number of members of the group who participate as co-authors in the *i*-th article. The index *IC* is:
	$\mathrm{IC}=\frac{\sum_{i=1}^{N}A_{j}}{N}-1$

The Lawani and Subramanyam indicators were used as indicators of both national and international collaboration. The Lawani indicator is typically employed to define the number of authors per article (Morgado-Gallardo et al., 2018; Ravelo-Contreras et al., 2016; Ravelo-Contreras et al., 2020), while the Subramanyam indicator has been used to indicate the degree of international collaboration (León-Cano et al., 2022). In the present study, both indicators have been used broadly to define collaboration at the individual, institutional, and country levels, allowing for comparisons with previous studies.

To measure collaboration according to the Minciencias criteria, the cooperation (Icoop) and cohesion (IC) indicators were employed. The former measures the degree of collaboration between groups, corresponding to the Lawani index, which considers Minciencias groups as entities, from which 1 is subtracted. On the other hand, the cohesion index measures the degree of collaboration within each group, considering the average number of individuals from each group who participate in the group’s articles, minus 1.

To describe the increasing or decreasing trend of a specific indicator over the period studied, the average annual growth rate (AAGR) was calculated. This indicator has been used in bibliometric studies in fields such as economics (Ho et al., 2022) and health sciences (Romanini et al., 2021), when the goal is to provide a quantitative measure of trends over relatively long periods of time. In addition, we estimated whether this trend was significant using the nonparametric Mann-Kendall monotonic trend test (Meals et al., 2011).

The population was Colombian psychology articles published during 2014–2023 in journals indexed in Web of Science, SciELO, and Scopus. Inclusion criteria were: (a) the journal where the article was included had psychology as its subject; (b) in journal articles with miscellaneous topics, the content of the article reviewed manually was psychology; (c) the article was a primary source research text (empirical or theoretical study) or secondary source (narrative or systematic review); and (d) at least one author of the article had at least one Colombian affiliation. As exclusion criteria, it was considered that the article was not a primary or secondary source study, such as editorials, book reviews, letters to the editor, etc.

The collection of articles was performed by combining the results of the web interface for Scopus and Web of Science, as well as direct database access via Python for SciELO and Scopus, using the packages *articlemetaapi* (Batalha, 2016) and *pybliometrics* (Rose & Kitchin, 2019), respectively. The collection process started on March 3, 2024, and was concluded on April 8, 2024. Once the information for all the databases had been collected separately, a careful process of standardization of information was carried out by creating two thesauri, one for authors and the other for affiliations, with the aim of correcting errors and eliminating duplicates.

Once the databases were corrected separately, they were all transformed into a common database, eliminating duplicate articles. For this, an adaptation of the method of Jiang et al. (2014) for bibliographic articles and the use of blocks described by Hadzic and Sarajlic (2020) were used. Specifically, we searched for duplicate titles within each database, keeping the version with the oldest year. For each article in a database, we searched for possible duplicates in the others, considering as criteria the DOI, the year of publication, the presence of the same publication meta-information (journal, volume and pages) and the title, both identically written and those written in a similar way. The presence of duplicate information for different versions of the same article made it possible to detect inconsistencies, thus improving the thesauri of authors and affiliations.

At the end of the integration process, a total of 4,686 unique documents were collected. To ensure that the articles were relevant to psychology, a supervised machine learning system was used to determine which articles were not pertinent after a manual review of a larger database of 11,100 articles with Hispano-American affiliation, performed by the lead author. According to this criterion, 490 articles were eliminated, with 4,196 articles ultimately being considered.

Specific procedures were established to identify the gender of the authors; the number of citations per article; the composition of the Minciencias groups; the research communities with which each researcher is associated; and, finally, the affiliation and main country for each researcher. In the case of gender, information from the Namsor gender identification service (Namsor Applied Onomastics, 2023) was used, as well as a custom-trained machine learning system. In the case of citations per article, the maximum number of citations reported by the Web of Science, Scopus and SciELO databases was considered as an indicator, in addition to the information available in Crossref for articles with DOI. This is consistent with the recommendation to combine information from multiple databases to obtain a reliable measurement of citations, as differences in the coverage of each database result in specific biases (Anker et al., 2019).

To determine the Minciencias research groups of the researchers, the complete information of the Minciencias groups and their members was downloaded from the site Science and Technology for All (Ministerio de Ciencia Tecnología e Innovación [Ministry of Science, Technology and Innovation], 2024). An association was made between this information and that available in the databases using the name of the researcher, as well as other identifiers (ORCID, Scopus and Web of Science).

To define the affiliation and main country of the authors, the fractional count was used (Perianes-Rodriguez et al., 2016). For each author, the scores for all affiliations and countries in the total number of publications in which he/she participated were summed. The affiliation and country with the highest score for each author was considered as his or her main affiliation and country, respectively.

## Results

### Productivity

**Figure 1.**
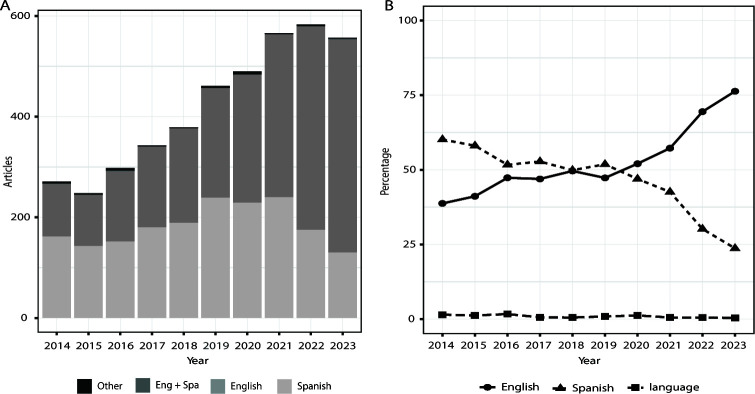
Distribution of papers by language. Panel A: Absolute number of papers among Spanish, English, both Spanish and English, and other languages. Panel B: Percentage of papers in Spanish, English, and other languages relative to the total.

The analysis considered a total of 4,196 Colombian psychology articles published in 2014–2023, published in the Web of Science, Scopus, and SciELO databases. Figure 1 shows a significant growth in the number of articles published per year, AAGR *=* 8.8%, *p <* .001, from 271 in 2014 to 557 in 2023. In the articles published in only one language, the majority are in English (*n =* 2314, 55.1%) and in second place in Spanish (*n =* 1,839, 43.8%). Both the number and the proportion of articles that have an English version have progressively increased, this increase being statistically significant, AAGR *=* 17.4%, *p <* .001. The number of articles published in Spanish per year has remained practically constant over the period, with a AAGR *=* –.8%, which is not significant, *p =* .371. In proportional terms, the number of articles published in Spanish per year has decreased, from 6.1% of the total number of articles published in 2014 to 23.7% in 2023.

**Figure 2.**
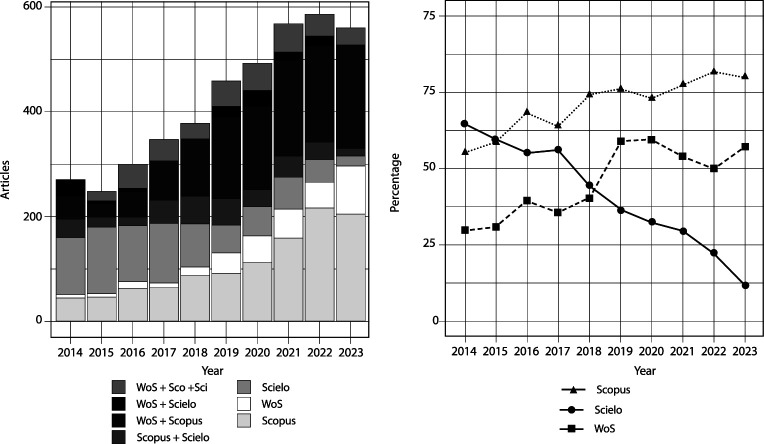
Distribution of papers across bibliographic databases. Panel A: Article coverage by each database and their overlap. Panel B: Percentage coverage of Scopus, Web of Science, and SciELO relative to the total.

With respect to the bibliographic databases, a total of 2,030 articles were incorporated from Web of Science, 3,076 from Scopus, and 1,546 from SciELO. Regarding the overlap, 2,134 articles (5.9%) were included in a single database, 1,668 (39.7%) in two, and 394 (9.4%) in all three databases. The number of articles indexed in Web of Science has increased significantly, AAGR = 19.1%, *p <* .001, as well as in Scopus, AAGR = 13.8%, *p <* .001. In contrast, the absolute number of articles in SciELO remained relatively constant between 2014 and 2021, with a slight average annual decrease, AAGR = –7.8%, which is not statistically significant, *p* = .088. These trends can be viewed in *Figure 2*.

With respect to the authors of the articles and their main country of affiliation, a total of 15,099 authors with affiliations in 134 countries, including Colombia, were identified. Of the total number of authors, 7,937 were women and 7,152 were men. Table 2 shows that the authors associated with Colombia as the main country were 5,752, of which 3,438 were women and 2,314 were men. The countries with the greatest collaboration with Colombian psychology, with more than 200 articles in the period, were Spain, the United States, Chile, Mexico, Brazil, the United Kingdom, Argentina, and Italy. Spain is the country with the highest number of articles co-authored with Colombian authors, and the United States is the country with the highest number of authors associated with Colombian productivity in psychology in the period.

**Table 2:** Articles and Authors Affiliated with Colombia and Top Collaborating Countries with the Highest Publication Counts (2014–2023)

Country Affiliations	Articles	Authors grouped by Country of Main Affiliation		
		Total	Male	Female
Colombia	4.196	5.752	2.314	3.438
Spain	812	1.104	574	530
United States	642	1.561	821	740
Chile	349	352	196	156
Mexico	289	318	149	169
Brazil	277	373	174	199
United Kingdom	256	403	214	189
Argentina	231	275	133	142
Italy	222	238	109	129
Total	4.196	15.099	7.152	7.937

A total of 4,407 affiliations were identified, of which 657 (14.9%) were Colombian. Table 3 displays the Colombian affiliations with more than 100 articles published during 2014–2023. Notably, all 12 affiliations are university institutions, eight of which offer doctoral programs. Of these, seven are certified with Qualified Registration, indicating that their programs meet the minimum quality standards required by the Ministry of Education, and one holds High-Quality Accreditation. Among these 12 affiliations, 10 show statistically significant annual growth in the number of published articles during 2014–2023.

**Table 3:** Number of Articles, Number of Authors, and Annual Growth Rate (AAGR) of Articles for Colombian Affiliations with the Highest Publication Counts (2014–2023)

Affiliation	Doctoral program	Articles	Authors	AAGR	*p-*value ^b^
Universidad Nacional de Colombia [National University of Colombia]	Qualified Registration	1179	1335	9.9	.001
Universidad de San Buenaventura [Saint Bonaventure University]	Qualified Registration	630	707	1.3	<.001
Universidad [Cooperative Cooperativa University of de Colombia] Colombia	No	391	362	19.5	.127
Pontificia Universidad Javeriana [Pontifical Javeriana University]	Qualified Registration	380	392	6.1	.105
Universidad del Norte [Northern University]	Qualified Registration	222	199	14.7	<.001
Universidad CES [CES University]	No	181	224	13.3	.0153
Universidad [Pontifical Bolivarian Pontificia University] Bolivariana	No	149	166	11.6	.0035
Universidad de Manizales [University of Manizales]	Qualified Registration	145	177	4.3	.0012
Universidad University] de la Costa [Coastal	No	143	84	46.3	.0123
Universidad del Valle [Valley University]	High-Quality Accreditation	129	127	19.9	.0107
Universidad Católica Luis Amigo [Luis Amigo Catholic University]	Qualified Registration	126	142	31.3^a^	^a^ .0327
Universidad de la Sabana [University of the Sabana]	Qualified Registration	108	163	12.4	.0191

*Note. ^a^ AAGR calculated for 2016–2024, as no articles were published in 2015. ^b^ Mann-Kendall nonparametric trend test.*

Regarding the Minciencias groups, a total of 1,829 groups associated with the authors who have published articles in psychology in the period were identified. Among these groups, only 149 are officially classified within knowledge areas directly associated with psychology, while the remaining 1,680 are classified in 150 other areas. The total number of authors affiliated with Minciencias groups is 2,509, of whom 2,361 have Colombia as their primary affiliation, accounting for 94.1% of the total. Table 4 displays the top 10 Minciencias groups with the highest number of authors, of which nine belong to the A1 category, denoting the highest productivity, and six are classified within psychology-related areas. Among these 10 groups, four exhibit statistically significant annual growth in the number of published articles within the 2014–2023 period.

**Table 4:** Minciencias Groups with the Highest Number of Authors

Minciencias Group	Ranking	psychology Area is	Articles	Authors	AAGR	*p*-value ^j^
Grupo de Investigación en Cien- cias del Comportamiento [Behavioral Sciences Research Groupa]^a^	A1	Yes	43	226	25.46	.127
Estudios Clínicos y Sociales en Psicología [Clinical and Social Studies in Psychology]^b^	A1	Yes	37	188	21.99	.037
Grupo de Neurociencias de Antioquia [Antioquia Neuroscience Group]^c^	A1	No	37	104	14.38	.589
Psicología del Consumidor [Consumer Psychology]^d^	A1	Yes	36	169	12.19	.085
Grupo de Investigación de Psicología [Psychology Research Group]^e^	A1	Yes	36	101	111.2	.124
Grupo de investigación en psicología cognitiva [Cognitive psychology research group]^f^	A1	Yes	34	73	14.26	.030
Estudios de Fenómenos Psicosociales [Studies of Psychosocial Phenomena]^g^	A	No	34	99	56.63	.019
Grupo de Investigaciones En Desarrollo Humano [Human Development Research Group]^h^	A1	No	31	120	9.943	.037
Grupo Neuropsicología y Conducta [Neuropsychology and Behavior Group]^i^	A1	No	31	100	9.88	1
Europsis	A1	Yes	31	151	2.695	.084

*Note. ^a^Research Group in Behavioral Sciences. ^b^ Clinical and Social Studies in Psychology. ^c^Neurosciences Group of Antioquia. ^d^Consumer Psychology. ^e^Psychology Research Group. ^f^Research Group in Cognitive Psychology. ^g^Studies of Psychosocial Phenomena. ^h^Human Development Research Group. ^i^Neuropsychology and Behavior Group. ^j^Mann-Kendall nonparametric trend test.*

### Collaboration

During the period under study, there was an increase in collaboration, in terms of authors, affiliations, and participating countries per article, as shown in Table 5. The Lawani collaboration index corresponds to the average number of entities per article. In 2014–2023, the Lawani index of authors was 6.6, that of affiliations 4.6 and that of countries 2.9. There was an increasing trend for the Lawani index of authors, AAGR = 9.4%, *p =* .004, as of affiliations, AAGR = 11.2%, *p =* .002 and of countries, AAGR = 6.8%, *p =* .002. Another indicator of interest is the Subramanyam index, which measures the proportion of articles with two or more authors. During the period studied, the Subramanyam index for authors was .90, for affiliations .66, and for countries .49. A sustained increase per year is observed for authors, AAGR = .6%, *p =* .07, affiliations, AAGR = 3.8%, *p =* .002, and countries, AAGR = 3.2%, *p =* .004. It is relevant to note that, starting from 2020, the proportion of articles in collaboration with other countries exceeds 50%, reaching a maximum of 55% in 2022.

**Table 5:** Indicators of Collaboration in Colombian Production in Psychology, 2014–2023

Indicator	2014	2023	Total	AAGR	*p-*value^a^
		*Lawani*			
Author	4.54	8.36	6.56	9.358	.004
Affiliation	2.93	6.16	4.59	11.24	.002
Country	2.23	3.57	2.87	6.757	.002
		*Subramanyam*			
Author	.87	.92	.90	.6	.074
Affiliation	.54	.74	.66	3.83	.002
Country	.41	.53	.49	3.244	.004

*Note. ^a^ Mann-Kendall nonparametric trend test.*

Our analysis explored whether the increase in collaboration stemmed from greater local and/or international involvement. The results showed higher collaboration indices for authors with foreign affiliations (average Lawani index of 4.15) compared to those with Colombian affiliations (average 2.41). The number of foreign authors per article grew annually by 18.4% (*p =* .007), while the number of Colombian authors per article grew slowly (AAGR = 2.1%, *p =* .032). A similar trend was observed in the Lawani index of affiliations, with foreign affiliations per article increasing by 19.03% annually (*p =* .002), and Colombian affiliations showing little change (AAGR = 3.16%, *p =* .003). Within Colombian collaboration, focusing on Minciencias groups, 3,334 articles featured at least one author from these groups, with a high general collaboration level indicated by a Lawani index of 3.6 and a Subramanyam index of .77; no significant trends were noted in these indices during the period analyzed.

Using the indicators defined by Minciencias to evaluate collaboration among the Minciencias groups, the mean of the cooperation indicator (Icoop) for the groups studied is M = 5.0 (SD = 2.5). The minimum value observed is 0, while the maximum value is 23, indicating a high degree of cooperation among the groups. Additionally, the cohesion index, which measures the degree of participation of members within the same group in the articles, has a mean of .13 (SD = .34). The minimum value recorded is 0, while the maximum value is 5. These findings suggest that the articles were produced by a reduced number of members within each Minciencias group.

### Internationalization

To evaluate the internationalization of Colombian psychology during the specified period, we considered publications in both Colombian and foreign journals, along with the number of citations per year based on the nationality of the main authors’ affiliations, the country of publication, and the availability of an English version. Colombian psychology articles have been published in a total of 702 journals from 2014 to 2023. Of the total number of articles, 1,511 have been published in Colombian journals, accounting for 36.01% of the total.

When analyzing the country of publication for journals associated with Colombian psychology during this period, Table 6 reveals a sustained growth in the number of articles published in foreign journals. Notably, there has been significant growth (19.6%, *p =* .001) in publication in U.S. journals, surpassing the number of publications in Colombian journals in the year 2023, with 124 articles versus 113. Furthermore, significant annual growth rates have been observed for journals published in England (27.1%), Spain (2.7%), Switzerland (25%), Brazil (15%), the Netherlands (21.1%), Latin America in general (3.5%), Europe (3.6%), and Argentina (39.5%).

**Table 6:** Number of Articles with Colombian Affiliation, Number of Journals, and Annual Growth in the Number of Articles, According to the Country of Publication

Country	Articles	Magazines	AAGR	*p-*value ^a^
Colombia	1.511	24	–3.6	.928
United States	714	254	19.6	.001
United Kingdom	461	165	27.1	.001
Spain	352	47	2.7	.003
Switzerland	236	17	25	.002
Brazil	208	41	15	.008
Netherlands	192	45	21.1	.001
Argentina	118	6	39.5	.003
Chile	100	9	83.7	.088
Latin America	164	32	3.5	.007
Europe	126	47	3.6	<.001
Other	14	13	–	–

*Note. ^a^ Mann-Kendall nonparametric trend test.*

While the number of articles published per year in Colombian journals remained relatively constant between 2014 and 2021, there was a sharp decrease of 57% between 2021 (164 articles) and 2023 (113 articles). However, the overall decrease for the entire period is not statistically significant, with a TCAP of –3.6% and *p-*value of .928.

**Figure 3.**
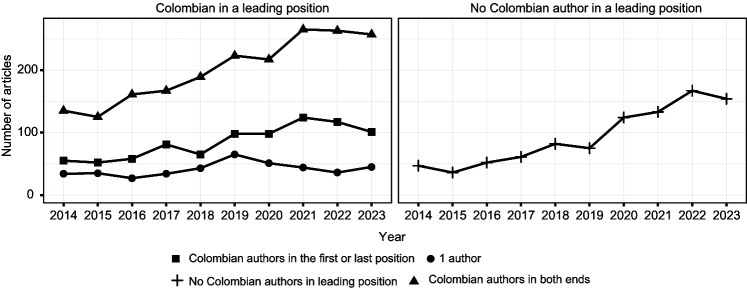
Number of articles per year, according to the presence or absence of Colombian authors in the main positions

From 2014 to 2023, a notable increase in international collaboration was observed, marked by a rise in the number of foreign authors and countries contributing to each article, as well as a higher proportion of articles involving foreign participation. The analysis focused on whether this rise was due to Colombian-led or foreign-led research. Using the number of articles with a Colombian as either the first or last author (key positions by convention) as an indirect measure, it was observed (*Figure 3*) that while articles by single authors (9.9%, all Colombian due to study criteria) remained steady (AAGR = 6.1%, *p =* .088), articles with both key positions held by Colombians (47.7% of total) grew at an annual rate of 8.08% (*p =* .002). Similarly, articles with one Colombian in these key roles (2.2% of total) increased by 9.33% annually (*p =* .003), while those with no Colombians in these

positions (22.2% of total) experienced the most rapid growth, surging by 17.18% annually (*p <* .001).

**Figure 4.**
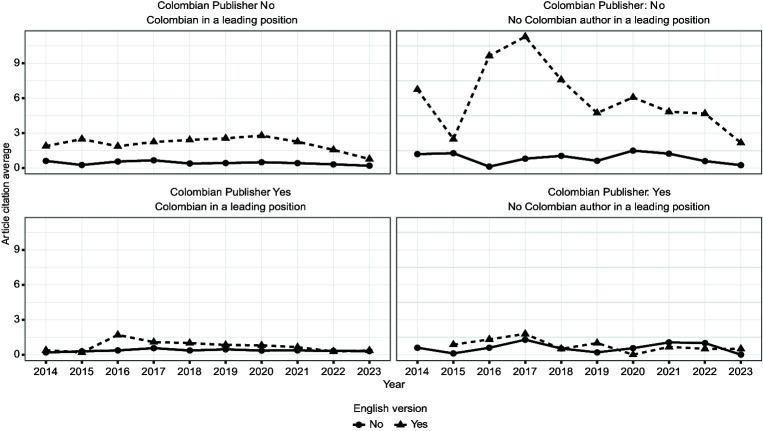
Average number of citations per article, according to the country of the scientific journal, nationality of the main authors, and language of the article

*Figure 4* presents the average number of citations per year for articles, considering factors such as the country of publication (Colombia or another country), the presence or absence of an English version, and whether a Colombian researcher holds a leading position. Using the Mann-Whitney U test, a significant difference is observed in the number of citations in favor of articles with an English version (M = 14.3, Med = 4) compared to those without (M = 2.02, Med = 1), U = 1211312, *p <* .001. Articles with a foreign author in leading positions (M = 23.32, Med = 8) have significantly more citations compared to those with Colombian authors in leading positions (M = 4.68, Med = 1), U = 781731, *p <* .001. Moreover, articles published in foreign journals (M = 12.5, Med = 3) receive significantly more citations than those published in Colombian journals (M = 2.48, Med = 1), U = 2776499, *p <* .001. Regarding the trend throughout the period, no statistically significant increases or decreases were observed in the number of citations according to the specified criteria.

## Discussion

The present study aimed to analyze the production of Colombian psychology from 2014 to 2023, utilizing data from three bibliographic databases (Web of Science, Scopus, and SciELO). The study focused on examining productivity, internationalization, and collaboration. The main findings reveal an increase in productivity, particularly in English, as well as collaboration with authors affiliated with foreign institutions. Notably, the level of cooperation and cohesion among groups within Colombia remained consistent.

There is a sustained annual growth in the number of articles published in English, while the proportion of articles published in Spanish has been decreasing in relation to the total. This contrasts with the trend observed in the mid-2010s, when most articles were published in Spanish (Ávila-Toscano & Marenco-Escuderos, 2016; Ravelo-Contreras et al., 2016). León-Cano et al. (2022) highlight that journal articles in Spanish without international collaboration receive a low number of citations. This finding aligns with the results of our study, that the most cited articles are those published in English, authored by individuals from foreign institutions in leading positions, and published in foreign journals.

International collaboration has great relevance in the field, both in terms of the number of authors involved and its growth. Approximately 61.5% of the authors contributing to the Colombian literature in psychology are foreigners, with a significant representation from affiliations in Spain and the United States. This finding aligns with previous literature (Cudina & Ossa, 2016; Gallegos et al., 2020; Ravelo-Contreras et al., 2016). The Subramanyam index for countries during the period stands at .49, which is very similar to the .47 previously reported in a study solely conducted using Scopus (León-Cano et al., 2022).

In terms of the type and productivity of the main Colombian affiliations, all 12 Colombian institutions with more than 100 articles during the period are universities, each showing positive annual growth in their publications. The presence of eight universities with doctoral programs underscores the pivotal role of these institutions in driving the growth of Colombian psychology’s productivity, as noted by López-López et al. (2022). These universities act as engines of scientific production, generating high-quality knowledge and strengthening national and international collaboration networks.

Regarding collaboration between affiliations, there is a high degree of international cooperation, with only 14.9% of the 4,407 identified affiliations being Colombian. Among the foreign affiliations, those from Spain, the United States, Argentina, Italy, and Chile have the highest number of associated articles. A similar pattern of collaboration is evident in the field of health psychology, as reported by Salamanca-Camargo & Tovar-Gamboa (2022).

Although, globally, most articles from the period were led by Colombian authors (77.8% vs. 22.2%), the proportion of articles directed by authors with foreign affiliations has experienced significant annual growth of 17.2%, surpassing the growth rate of articles led by Colombian authors, which stands at 7.4%. In 2014, 17.3% of projects were led by international authors, compared to 27.6% of articles in 2024. This trend can be viewed positively, as it reflects the interest of international groups in integrating Colombian academics, which diversifies the productivity of Colombian psychology (López-López et al., 2022). However, it is essential to consider that artificially spurred growth in productivity, driven by external pressures, such as the pursuit of internationalization, could weaken the study of national issues (Chinchilla-Rodríguez et al., 2015), which is one of the strengths of Colombian psychology (Vesga, 2021).

Several indicators demonstrate that researchers and research groups have adapted to the Minciencias evaluation criteria associated with scientific productivity (Ministerio de Ciencia, Tecnología e Innovación [Ministry of Science, Technology and Innovation], 2020b). Firstly, there is a high level of cooperation among the Minciencias groups, with an average score of 4.9. Conversely, the level of cohesion is relatively low, with an average of .13. This could be attributed to the emphasis placed on the criterion of cooperation over cohesion in the evaluation process. Secondly, the publication rate has shown an increase, particularly in foreign journals indexed in Web of Science and Scopus. This trend aligns with the encouragement to publish in high-impact journals, as promoted by the measurement model.

The strengths of this study lie in the utilization of multiple databases and a rigorous data-cleaning process, which instills confidence in the validity of the analyzed data. This enables the provision of indicators that can be utilized in future studies to assess advancements in productivity and internationalization of Colombian psychology. Furthermore, this study represents the first bibliometric analysis that incorporates the Minciencias groups into the evaluation of productivity within the field of psychology in Colombia.

## Conclusion

This paper explores how Minciencias policies, especially the measurement and recognition model, align with academic group strategies, affecting academic production as shown through bibliometric analysis (Pérez-Martelo et al., 2022). The results provide evidence of the consolidation of psychology in Colombia, as the increase in overall productivity, promoted by national policies (Romero-Torres et al., 2013), is sustained by university institutions, primarily those associated with doctoral programs (López-López et al., 2022), as well as by the highest-quality A1-category Minciencias research groups. This development has been largely achieved through collaborative efforts, both locally and internationally (Puche-Navarro et al., 2022). The internationalization of Colombian psychology can now be affirmed as a reality, given the high level of international collaboration and the publication of research in international journals and in English, which enhance the visibility of work by Colombian authors. However, a concerning issue is the stagnation in local journal publications, despite the growth in international journals, as this could limit the visibility of context-specific research, weaken local academic ecosystems, and reduce opportunities for emerging researchers.

Future research should compare these findings internationally to gauge whether the trends in internationalization and collaboration reflect broader disciplinary trends or are unique to Colombia. It is crucial to assess how the internationalization of Colombian research, driven by the increase in articles by foreign and mixed authorship, impacts the focus on locally relevant topics (Chinchilla-Rodríguez et al., 2015).

## Limitations

The primary limitation is the potential publication bias due to the exclusion of articles not included in the three databases. However, it is important to note that we utilized the databases recognized as the most relevant and that have been employed in previous studies (Cudina & Ossa, 2016; León-Cano et al., 2022). In the examination of internationalization, another limitation is the inability to differentiate between citations from articles authored by Colombians versus those by foreign authors. This distinction would have allowed for a more precise assessment of areas or topics with local versus international impact.
